# A prospective study of a training program for bronchial sleeve resection using operable 3-dimensional models

**DOI:** 10.1016/j.xjtc.2024.07.003

**Published:** 2024-07-31

**Authors:** Kohei Hashimoto, Daiki Kato, Junji Ichinose, Yosuke Matsuura, Masayuki Nakao, Sakae Okumura, Haruhiko Kondo, Takashi Ohtsuka, Mingyon Mun

**Affiliations:** a Department of Thoracic Surgical Oncology, Cancer Institute Hospital, Japanese Foundation for Cancer Research, Tokyo, Japan; b Department of Thoracic Surgery, Kyorin University, Tokyo, Japan; c Department of Thoracic Surgery, Jikei University, Tokyo, Japan

**Keywords:** 3D model, surgical education, bronchoplasty, lung cancer

## Abstract

**Objective:**

To develop a training program for bronchial sleeve reconstruction using our previously developed 3-dimensional (3D) operable airway model and evaluate its effectiveness in surgical trainees.

**Methods:**

Eight trainees and 4 faculty surgeons were enrolled. Their right upper lobe sleeve reconstruction procedures were scored by 2 senior surgeons in a blinded fashion on a 5-point Likert scale on the following: airway wall tear, reapplied ligatures, reapplied needles, needle entry and exit, anastomotic bite, and caliber adjustment (full score: 30). The trainees were randomized into training and control groups (n = 4 in each group). The training group underwent 6 cycles of training guided by video-based instructions. The control group underwent regular clinical training. All trainees were reevaluated.

**Results:**

Before training, the median score of faculty surgeons was better than that of trainees (27.0 [range, 21.0-28.0] vs 17.5 [range, 9.5, 26.5]; *P* = .05), suggesting the validity of the scoring method. The initial scores and anastomosis times were similar in the control and training groups. After training, the scores tended to be higher in the training than in the control group (median, 28.2 [range, 27.0-29.0] vs 20.8 [range, 15.0-28.0]; *P* = .11). The anastomosis time tended to be shorter in the training group (median, 20.0 [18.9, 21.6] minutes vs 24.6 [range 17.8-30.9] minutes; *P* = .69). The reduction in anastomosis time was significantly greater in the training group (median, −9.4 [range, −4.5 to −13.1] vs 0.0 [range, 5.3 to −6.0]; *P* = .05).

**Conclusions:**

The training program for bronchial sleeve resection using 3D airway models with video-based instructions improved the trainees’ skills.

## Graphical Abstract

**Figure:**
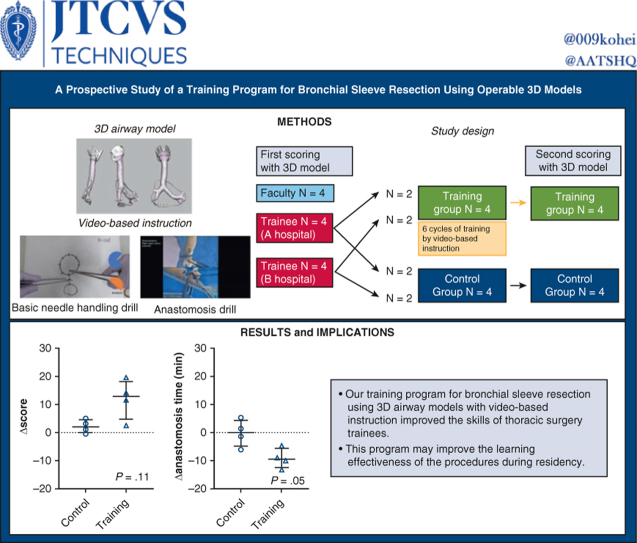

**Figure:**
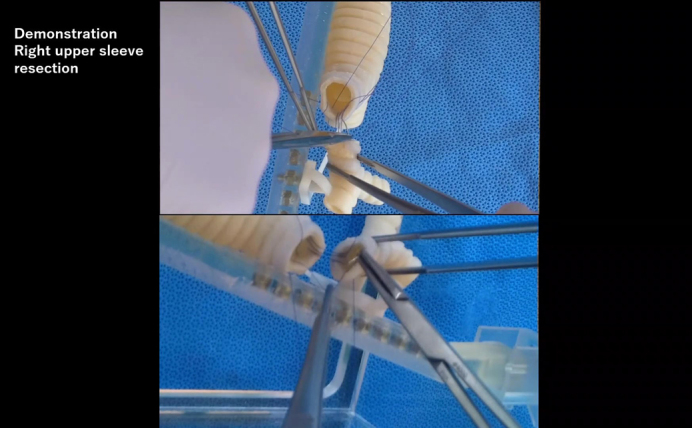
Bronchial sleeve resection training using 3D models improved the skills of trainees.

**Central Message**

Our training program for bronchial sleeve resection using 3-dimensional models improved the skills of thoracic surgery trainees. This training program may improve learning effectiveness during residency.

**Perspective**

Bronchoplasty skills are becoming increasingly difficult for thoracic surgery trainees to acquire through clinical experience. Our training program for bronchial sleeve resection using operable 3-dimensional models with video-based instructions improved trainees’ skills. This training program may improve the learning effectiveness of skills that cannot be adequately experienced during residency.

In lung cancer surgery, the number of cases requiring bronchoplasty has decreased in line with a decrease in the number of centrally located lung cancers.^1^^,^^2^ Nonetheless, bronchoplasty remains an important procedure for thoracic surgeons to master because of its potential impact on cancer curability and serious complications. The techniques involved in bronchoplasty also are required for lung transplantation^3^ or benign tracheobronchial diseases, such as central airway stenosis.^4^ However, the decreasing number of cases requiring tracheobronchial reconstruction has made it increasingly difficult for thoracic trainees to become proficient through their clinical experience alone. Failure to acquire this skill may result in significant disadvantages to patients, such as the inability to achieve complete resection, reluctance to operate on cases that normally would be resectable by thoracic surgery with sufficient tracheobronchoplasty technique, or complications due to lack of skill quality.

Complementary training methods exist, such as hands-on training with cadavers or porcine lungs. However, because these types of wet lab training require significant costs, dedicated time, including travel and preparation, and special facilities, they might not be sufficiently repeated to achieve proficiency. In addition, porcine lungs are anatomically different from the human lungs,^5^ and sacrificing live animals raises potential ethical issues. However, to the best of our knowledge, no dry lab training method dedicated to tracheobronchial reconstruction or anastomosis has been reported other than ours.^6^ A training method that can provide hands-on experience similar to the actual procedure and can be performed with flexible timing can fill this gap. Furthermore, a training method that is scientifically proven to be effective for thoracic surgical trainees is desirable.

In this study, we aimed to develop a training program for bronchial sleeve reconstruction techniques using our previously developed 3-dimensional (3D) operable airway models that sufficiently replicate surgical experience. We also evaluated the program's effectiveness on thoracic surgery trainees in a prospective study.

## Methods

### Candidate Selection

This prospective study was conducted at the Cancer Institute Hospital and the Jikei University Hospital. The study was approved by the Institutional Review Board of Cancer Institute Hospital (2020-GA-1334) on May 28, 2021, and consent was obtained from all participants.

**Figure 1:**
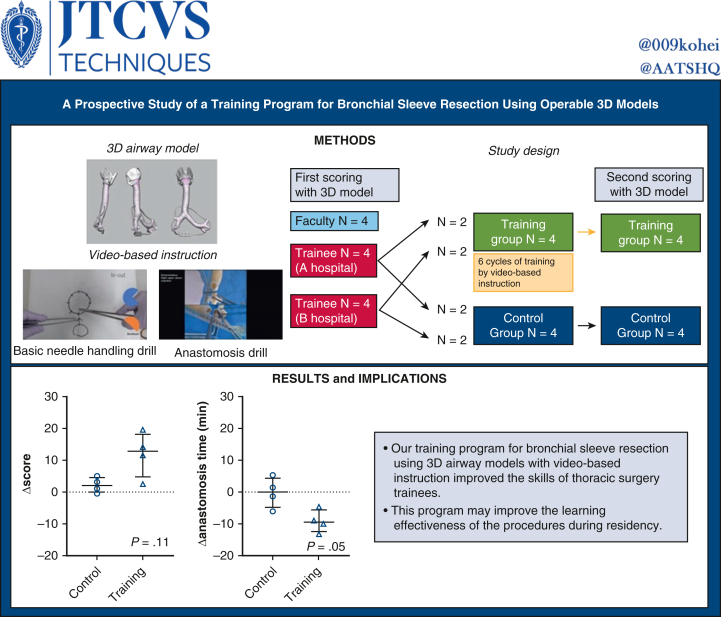
(Graphical abstract). Study design and main results. *3D*, 3-Dimensional.

The study design is illustrated in Figure 1. Four trainees and 4 faculty surgeons were recruited from the Cancer Institute Hospital. Four trainees were recruited from the Jikei University Hospital. Participants were informed that only the surgical field, without sound, was recorded and scored by blinded raters. After the first evaluation, the trainees were randomized into training and control groups (n = 4 in each group). Randomization was stratified by center (2 trainees in the control group and 2 in the training group in each center). The training group underwent 6 training sessions guided by video-based instructions (Videos 1 and 2) within 2 months. The control group received regular clinical training at each center. The second session was blinded as well. Although the 8 trainees were evaluated twice, the 4 faculty surgeons were evaluated only in the first session, because their technique was assumed to have plateaued for 2 months. Postgraduate year (PGY) was defined as the number of years after graduation from medical school (all participants were from Japanese medical schools), which included 2 years of general internships. The internship is now mandatory and does not focus solely on surgery in the Japanese system; therefore, actual surgical residency begins in the PGY 3. Dedicated research time (typically 2 to 3 years), which some trainees had, was not excluded from the postgraduate years. A Japanese surgical training program typically consists of 1 to 2 years of general surgery, followed by 3 to 5 years of thoracic surgery training. A cardiac rotation is optional. The length of thoracic surgery training varies widely from institution to institution. In addition, completion of training may be based on competency rather than on years of training.

### Airway Models and Surgical Instruments

**Figure 2:**
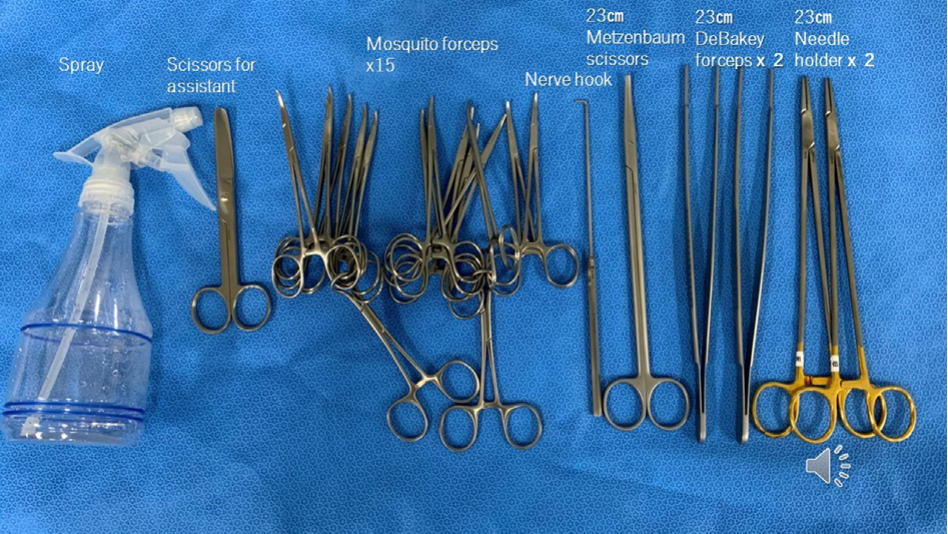
Surgical instruments used in the training.

The methods for creating the airway models used in this study have been described in one of our previous studies.^6^ In brief, this model was designed based on computed tomography data from a healthy volunteer. The model was made of structures of multiple stiffness (tracheobronchial rings and other parts) reinforced with thin gauze inside the body as cut resistance, In the previous study, board-certified thoracic surgeons found that this model satisfactorily replicates the surgical texture of the airway.^6^ The instruments used are shown in Figure 2. Two 23-cm fine-tip needle holders (TKX-HB2218; Takasago Medical Industry), two 23-cm Cooley forceps (TKZ8912-24; Takasago Medical Industry), 1 Metzenbaum scissors (TKZ-F2187-3; Takasago Medical Industry), 1 nerve hook (30-0300; Geister), 15 mosquito forceps (TKZ-E107; Takasago Medical Industry), 1 assistant's short scissors (TKZ-E101; Takasago Medical Industry), and a spray (daily use) for soaking the surgeon's hand with tap water before tying the knots. Double-armed 4-0 monofilament absorbable sutures with 20- to 22-mm needles (Maxon CV-24, 90 cm; Covidien Japan; PDSⅡSH-1, 90 cm; Ethicon) were used for anastomosis.

### Video-Based Learning of Bronchial Anastomosis

After the first assessment, the trainees assigned to the training group were provided the videos including the “basic needle handling drill” (Videos 1-4) and “anastomosis drill” (Videos 5-8), original content created for thoracic trainees based on our clinical experience and general knowledge. Before the first training session, the training group learned from the videos and could return to them repeatedly during the 6 training sessions.

### Scoring of the Techniques

**Figure 3:**
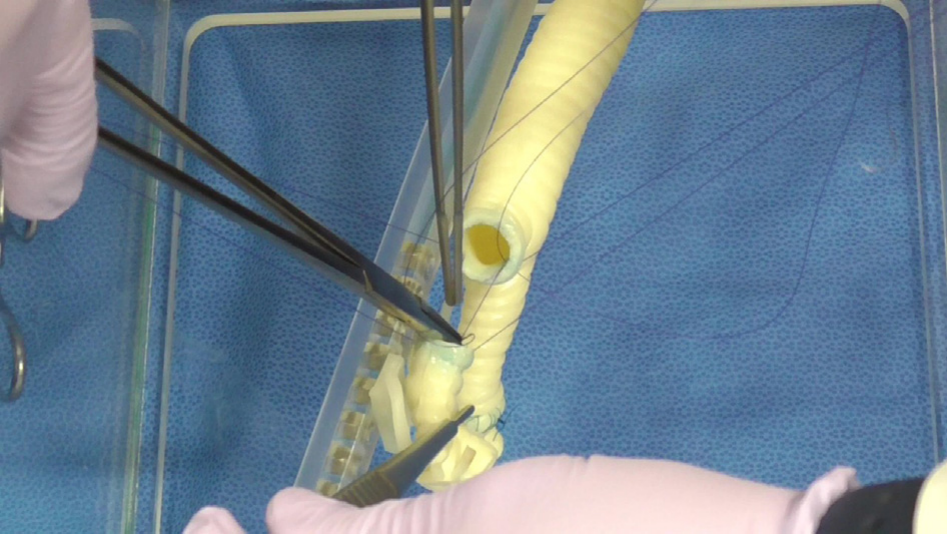
Recorded sleeve right upper lobectomy for blinded evaluation.

The right upper lobe sleeve reconstructions of the candidates were scored by 2 senior surgeons in a blinded fashion (Figure 3). The average score of the 2 surgeons served as the actual score for each evaluation. In the first session, the candidates were asked to perform an anastomosis as they would in daily clinical practice. In the second session, the candidates were asked to follow the same instructions as in the first session; however, the training group was also encouraged to incorporate the skills obtained from the training. The scoring sheets used in this study, shown in Table 1, and included a 5-point Likert scores for “airway wall tear,” “reapplied ligatures,” “reapplied needles,” “needle entry and exit,” “anastomotic bite,” and “caliber adjustment” (full score: 30 points). These terms were defined as follows: airway wall tear, the number of times the airway wall was cut or damaged by forceful needle or suture manipulation; reapplied ligature, the number of needle reapplications when needle placement was inadequate or suture was tangled; needle entry and exit, a subjective assessment of the angle of needle handling through the airway wall; anastomotic bite, a subjective assessment of the appropriateness of needle bite width; caliber adjustment, a subjective assessment of appropriateness of pitch adjustment for caliber discrepancy. The number of events was converted to a 5-point scale based on a preset conversion table (Table 1).

**Table 1:** Score sheet

Evaluation score	
Likert score	Poor moderate excellent
Number of cutouts	1 2 3 4 5
Number of reapplied ligatures	1 2 3 4 5
Number of reapplied needles	1 2 3 4 5
Needle entry and exit	1 2 3 4 5
Anastomotic bite	1 2 3 45
Caliber adjustment (pitch)	1 2 3 4 5

Score conversion from the number to the Likert score					
	1	2	3	4	5
Number of cutouts	2≦		1		0
Number of reapplied ligatures (incomplete knot is also counted)	3≦		2	1	0
Number of reapplied needles (releasing tangled threat is also counted)	4≦	3	2	1	0

Anastomosis time (minutes).

The anastomosis time (in minutes) was measured from the first touch of the model to the last touch of the model (including the suture attached to the model). To conserve resources, the process of cutting the bronchus was not included. Interrater reliability tests were performed on the first session scores from 8 trainees and 4 faculty members.

### Statistical Analysis

The Mann-Whitney *U* test was used to compare the characteristics and scores between the 2 study groups. The Fisher exact test was used to analyze categorical data, and the Wilcoxon signed-rank test was used to evaluate changes in the scores. All *P* values were 2-sided, and *P* < .05 was considered statistically significant. Statistical analyses were performed using Prism version 9 (GraphPad Software). Intraclass correlation coefficients (2, 1) of each scoring component of the 2 evaluating surgeons were calculated with 95% confidence interval for interrater reliability testing using SPSS version 25 (IBM).

## Results

### Characteristics of the Study Participants

The median postgraduate year (including 2 years of general internship before surgical residency and research time) of all trainees was PGY 8.5 (range, 7-12). One female trainee and 7 male trainees were recruited for this study. After randomization, the postgraduate years were similar between the control and training groups (median, 9 [range, 8-11] vs 7 [range, 7-12]; *P* = .31).

### Validation of Scoring by Comparing Faculty Surgeons and all Trainees

**Figure 4:**
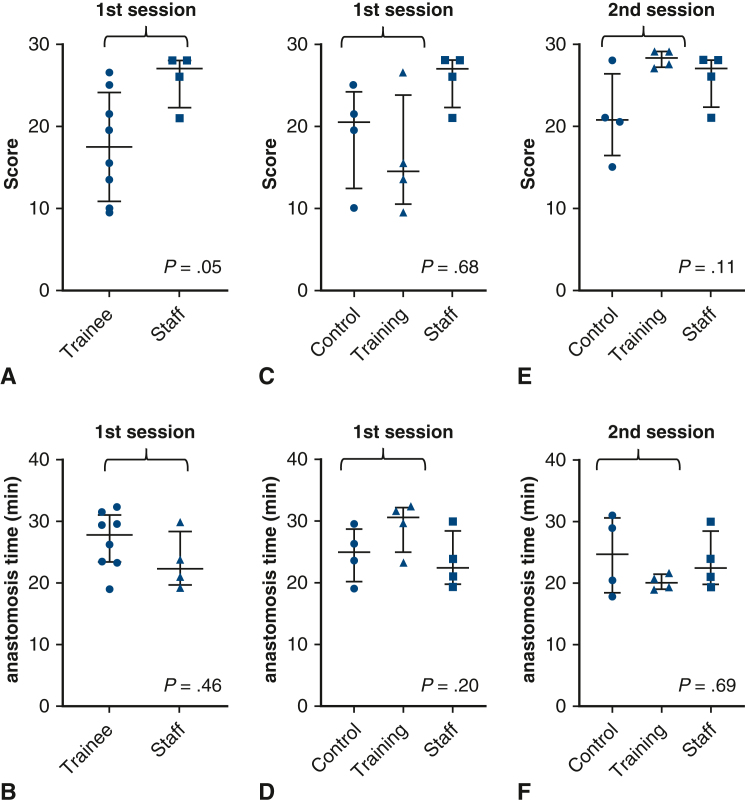
Comparison of anastomosis score and time at the first (before training) and second (after training) sessions. A, Scores of the trainees and staff at the first session. B, Anastomosis times of the trainees and staff at the first session. C, Scores of the control and training groups in the first session, with staff scores as the reference. D, Anastomosis times of the control and training groups in the first session, with staff scores as the reference. E, Scores of the control and training groups in the second session, with staff scores as the reference. F, Anastomosis times of the control and training groups in the second session, with staff scores as the reference.

In the first evaluation, the median score was significantly better for faculty surgeons compared to trainees (27.0 [range, 21.0-28.0] vs 17.5 [range, 9.5-26.5]; *P* = .05) (Figure 4, *A*), and the anastomosis time tended to be shorter among faculty surgeons than among trainees (22.4 [range, 19.4-29.9] minutes vs 27.9 [range, 19.1-32.5] minutes; *P* = .46) (Figure 4, *B*). These findings suggest the validity of our scoring methods by demonstrating the ability to discriminate between different groups. The intraclass correlation coefficient (2,1) in scoring of the first session was as high as 0.83, further supporting the validity of our scoring method.

### First Scoring and Anastomosis Time

After randomization, the first scores tended to be higher and the anastomosis time tended to be shorter in the control group compared to the training group (first scores: median 20.5 [range 10.0-25.0] vs 14.5 [range, 9.5-26.5]; *P* = .69; anastomosis time: median, 25.0 [range, 19.1-29.5] minutes vs 30.6 [range, 23.3-32.5]; *P* = .20). However, these differences were not statistically significant (Figure 4, *C* and *D*).

### Second Scoring and Anastomosis Time and Their Transitions

After training, the scores tended to be higher in the training group than in the control group (median, 28.2 [range, 27.0-29.0] vs 20.8 [range, 15.0-28.0]; *P* = .11), and the scores were similar to those of faculty surgeons (at the first session) (Figure 4, *E*). Anastomosis time also tended to be shorter in the training group (median, 20.0 [range, 18.9-21.6] minutes vs 24.6 [range, 17.8-30.9] minutes; *P* = .69) (Figure 4, *F*), and again, the time was close to that of the faculty surgeons.

**Figure 5:**
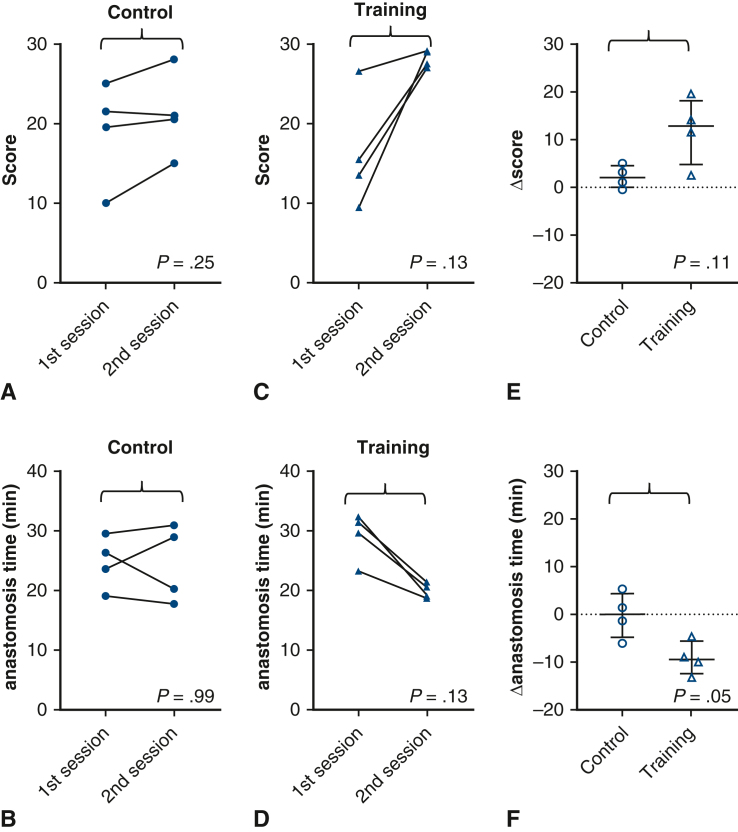
Transition of scores and times and changes (Δ) after training. A: Transition of the scores in the control group. B: Transition of the anastomosis times in the control group. C: Transition of the scores in the training group. D: Transition of the anastomosis times in the training group. E: Comparison of the change (Δ) in scores after training between the groups. F: Comparison of the change (Δ) in anastomosis times after training between the groups.

Although not statistically significant, the increase in scores from the first to the second session was more consistent in the training group (median difference, +12.8; *P* = .13; Figure 5, *A*) than in the control group (median difference, +2.0; *P* = .25; Figure 5, *C*). Similarly, the decrease in anastomosis time appeared more consistent in the training group (median difference, −9.4; *P* = .13; Figure 5, *B*) than in the control (median difference, +0.0; *P* = .99; Figure 5, *D*) group. The actual increase in score (Δscore) tended to be higher in the training group than in the control group (median, +12.8 [range, 2.5-19] vs +2.0 [range, −0.5 to 5.0]; *P* = .11) (Figure 5, *E*). The reduction in anastomosis time (Δanastomosis time) was significantly greater in the training group (median, −9.4 [range, −4.5 to −13.1] vs 0.0 [range, 5.3 to −6.0]; *P* = .05 (Figure 5, *F*).

## Discussion

In this prospective study on the efficacy of our training program on the bronchial sleeve resection technique for thoracic surgery trainees, the training program was found to significantly reduce the anastomosis time. There also was a clear trend toward improvement in the technical scores of the bronchial anastomoses. The uniqueness and strength of our study are based on a previously developed operable 3D airway model and our original instructional video materials. The simulation surgery on this 3D model was shown to be comparable to the clinical practice by experienced thoracic surgeons in our previous study.^6^ Trainees can learn and practice techniques in an environment similar to a clinical situation but without restrictions on time or place. This flexibility will be helpful for busy thoracic trainees.

Learning from videos potentially can be generalized anywhere in the world. Videos can be studied repeatedly, which is important for acquiring new knowledge and skills. The combination of our 3D model and video could be an instructive learning tool, particularly for areas without rich clinical training or many clinical experts in this tracheobronchoplasty technique. Technically, this method of using videos also has been thought to create less bias among trainees compared to in-person coaching in our study design. Nevertheless, the addition of in-person hands-on coaching of the anastomotic technique by experienced surgeons in each institution may further enhance learning opportunities for the actual use of our training method.

Currently, there is a growing need for simulation and off-the-job training in surgical education. Simulation training already has been formally integrated into surgical education for medical students in the United States.^7^ In a prospective survey of surgical trainees in England and Ireland, 98.9% of them felt that simulation training was important.^8^ At the international professional society level (European Society of Thoracic Surgeons), the need for simulation-based training in thoracic surgery is being actively assessed to establish systematic simulation training.^9^ There exist several types of surgical simulations, such as video-based learning,^10^, ^11^, ^12^ software-based or virtual simulation,^13^, ^14^, ^15^, ^16^, ^17^ and organ models such as ours. Few interactive 3D organ models have been reported to improve the quality of surgical simulations, including cardiac^18^ and brain^19^ surgeries. However, to the best of our knowledge, no systematic prospective study has investigated the efficacy of interactive 3D model simulators in any organ. We believe that quality assessment of training is important for surgical education tools, particularly when applied to complex procedures with considerable risks for the patient, such as tracheobronchoplasty.

Most surgical procedures for lung cancer do not require anastomosis or reconstruction. There is a clear contrast in the demand for needle-handling skills in thoracic surgery compared with cardiac surgery, which requires rapid and precise anastomosis. Historically, cardiac and thoracic surgeries have been performed by the same department or surgeons. More recently, they have tended to be separate divisions; however, this practice varies in different countries. The thoracic surgical training program may be combined with a cardiac program, as in North America, or isolated, as in Japan. Even in a combined cardiothoracic program, cardiac training might not be long enough for thoracic trainees to become proficient. Therefore, in some programs, the training of needle-handling skills in thoracic training programs might not be sufficient. Therefore, we also developed basic needle-handling skills, which are the foundation for high-quality airway anastomosis.

This study has some limitations, starting with the small sample size. The randomization with small sample size might not result in fully comparable cohorts. However, there was a clear trend of improvement in scores and a significant decrease in anastomosis time with our training method, even with a small sample size, and the initial score and time were favorable for the control group. We did not perform power calculations, mainly because of the challenge in estimating how the score would evolve, as the score was our original, in which there was no valid objective technical evaluation of this surgical technique. Second, other important components of tracheobronchial procedures were not trained in our methods, such as reducing tension (dividing the pulmonary ligament and cutting the pericardium) and avoiding excessive dissection of the peribronchial tissues. However, we believe that these components are less complex than anastomosis itself. Third, scoring might not completely reflect a reduction in complications after bronchial anastomosis. We developed our own scoring techniques that we believe are generally acceptable concepts for safe bronchial anastomosis; however, these scoring factors have not been validated to be directly related to the reduction of complications after bronchial anastomosis. Had we extended the training (for durability even with a cross-over design) or recruited more trainees, our results might have been stronger. We did not do this for practical reasons, such as limitation of limited resources and training duration. Nevertheless, we believe that a training program that can reduce anastomosis time and improve scores can be safely described as an effective educational method.

## Conclusions

Our training program for bronchial sleeve resection using 3D airway models with video-based instructions improved the skills of thoracic surgery trainees. This training program may improve the learning effectiveness of procedures that cannot be adequately experienced during residency programs.

## Conflict of Interest Statement

The authors reported no conflicts of interest.

The *Journal* policy requires editors and reviewers to disclose conflicts of interest and to decline handling or reviewing manuscripts for which they may have a conflict of interest. The editors and reviewers of this article have no conflicts of interest.

## Supplementary Data

Video 1 Basic needle handling drill. Part 1. Video available at: https://www.jtcvs.org/article/S2666-2507(24)00274-8/fulltext.

Video 2 Basic needle handling drill. Part 2. Video available at: https://www.jtcvs.org/article/S2666-2507(24)00274-8/fulltext.

Video 3 Basic needle handling drill. Part 3. Video available at: https://www.jtcvs.org/article/S2666-2507(24)00274-8/fulltext.

Video 4 Basic needle handling drill. Part 4. Video available at: https://www.jtcvs.org/article/S2666-2507(24)00274-8/fulltext.

Video 5 Anastomosis drill. Part 1. Video available at: https://www.jtcvs.org/article/S2666-2507(24)00274-8/fulltext.

Video 6 Anastomosis drill. Part 2. Video available at: https://www.jtcvs.org/article/S2666-2507(24)00274-8/fulltext.

Video 7 Anastomosis drill. Part 3. Video available at: https://www.jtcvs.org/article/S2666-2507(24)00274-8/fulltext.

Video 8 Anastomosis drill. Part 4. Video available at: https://www.jtcvs.org/article/S2666-2507(24)00274-8/fulltext.
