# Supplementary figures and images for: A prospective study of a training program for bronchial sleeve resection using operable 3-dimensional models
Source: JTCVS Tech. 2024 Jul 31;27:217–24. doi: 10.1016/j.xjtc.2024.07.003 (PMC11518966; doi:10.1016/j.xjtc.2024.07.003)

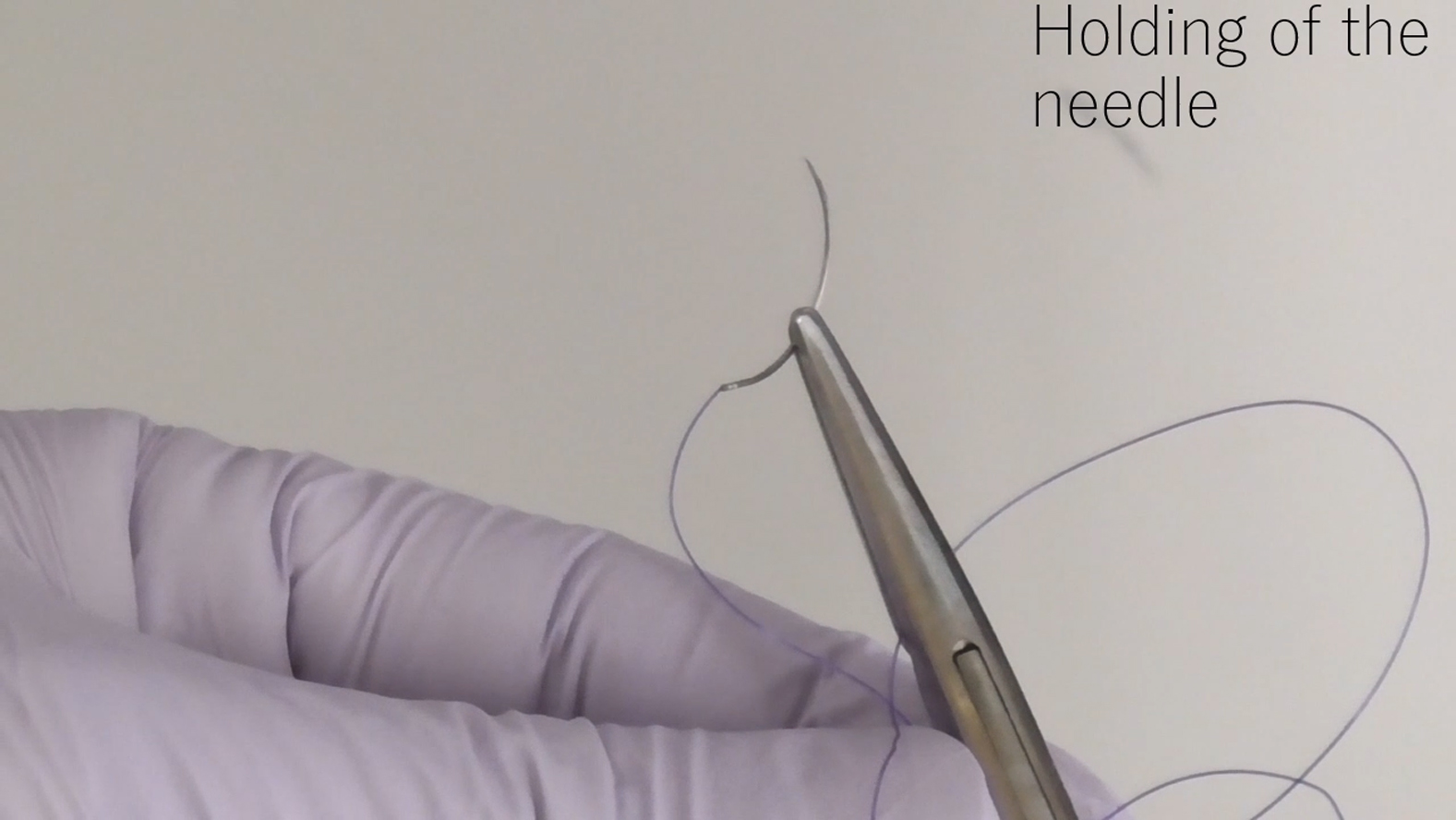

Supplement: Video 1 — Basic needle handling drill. Part 1. Video available at: https://www.jtcvs.org/article/S2666-2507(24)00274-8/fulltext. [file fx2.jpg]

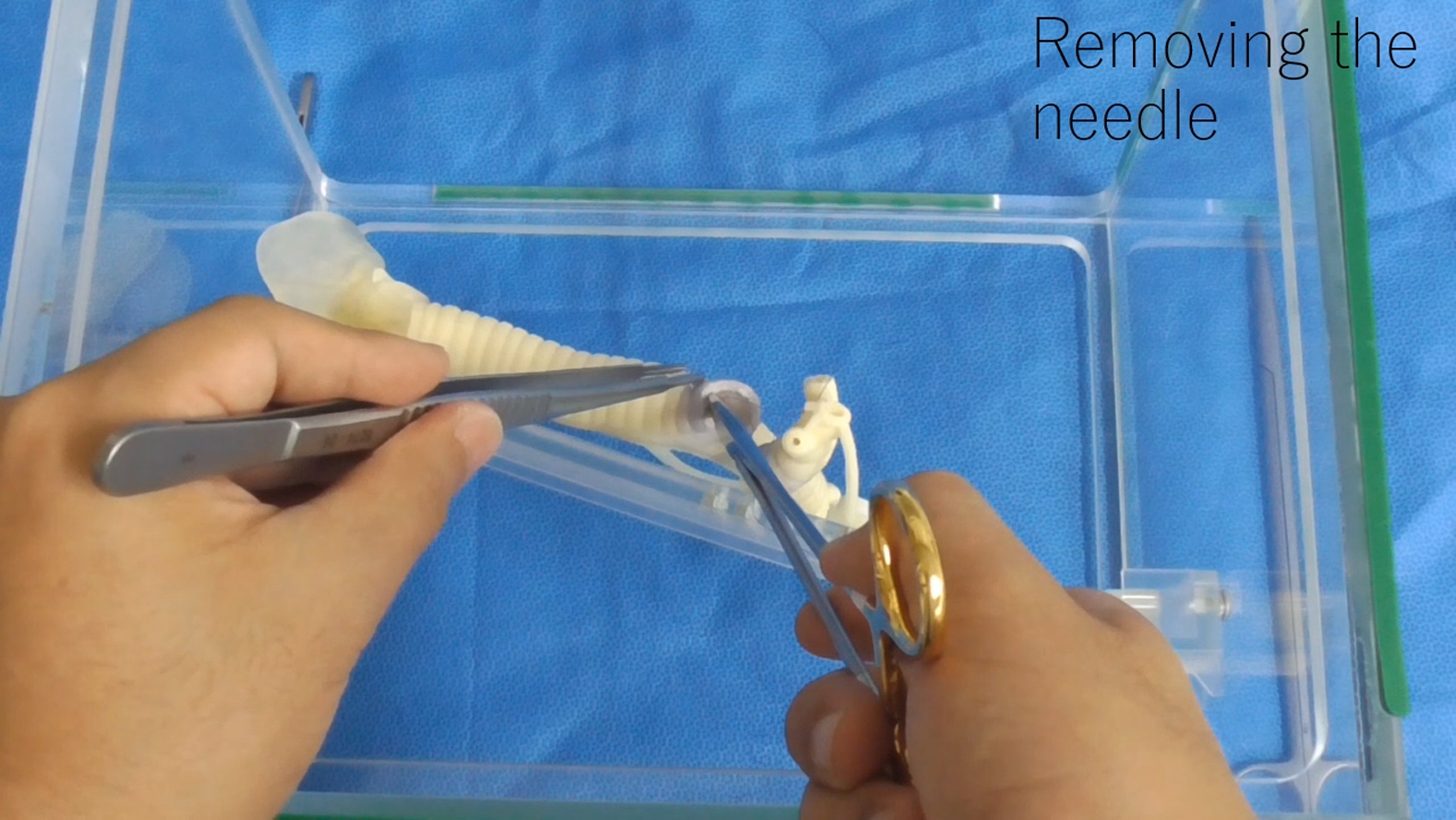

Supplement: Video 2 — Basic needle handling drill. Part 2. Video available at: https://www.jtcvs.org/article/S2666-2507(24)00274-8/fulltext. [file fx3.jpg]

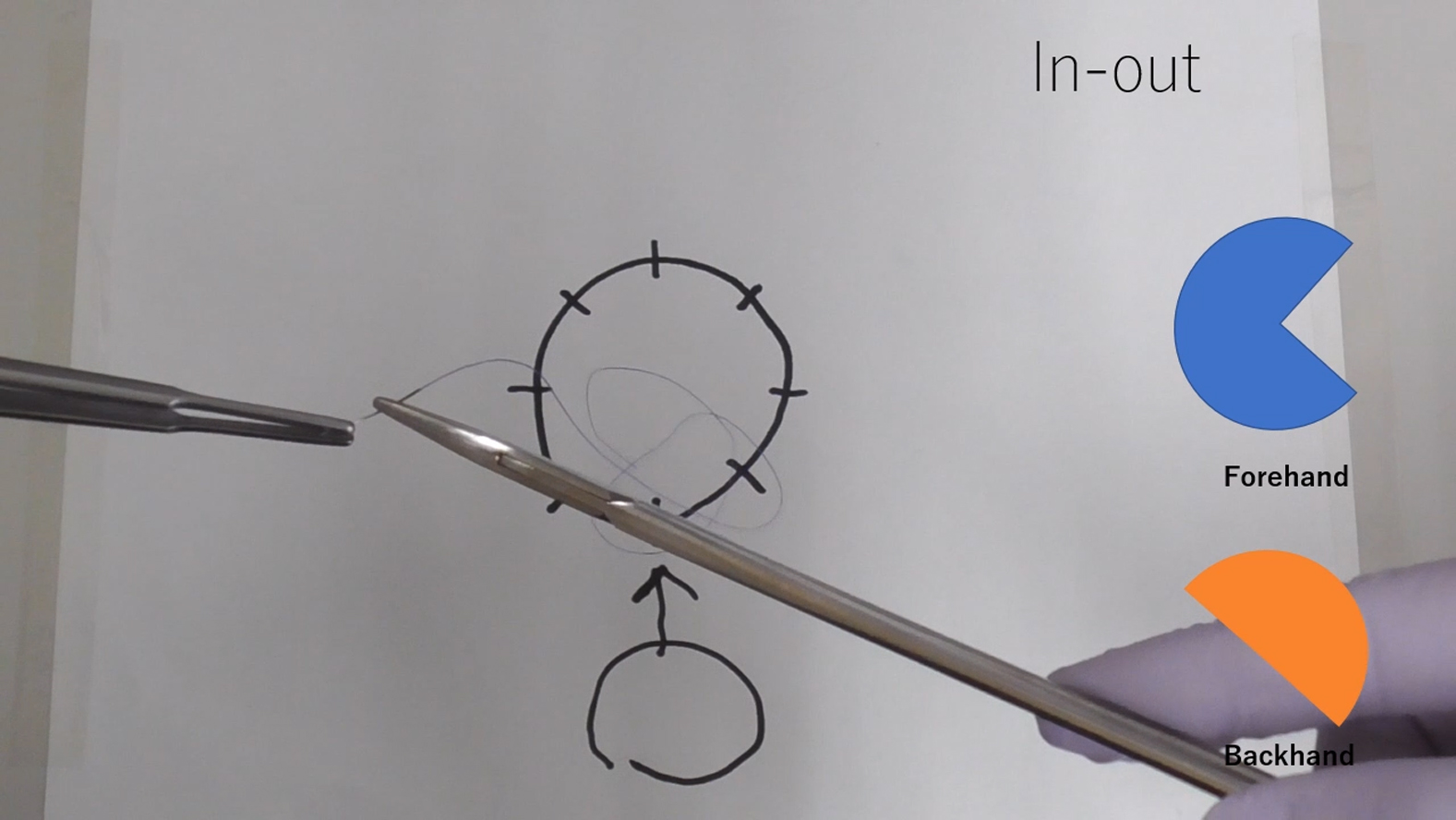

Supplement: Video 3 — Basic needle handling drill. Part 3. Video available at: https://www.jtcvs.org/article/S2666-2507(24)00274-8/fulltext. [file fx4.jpg]

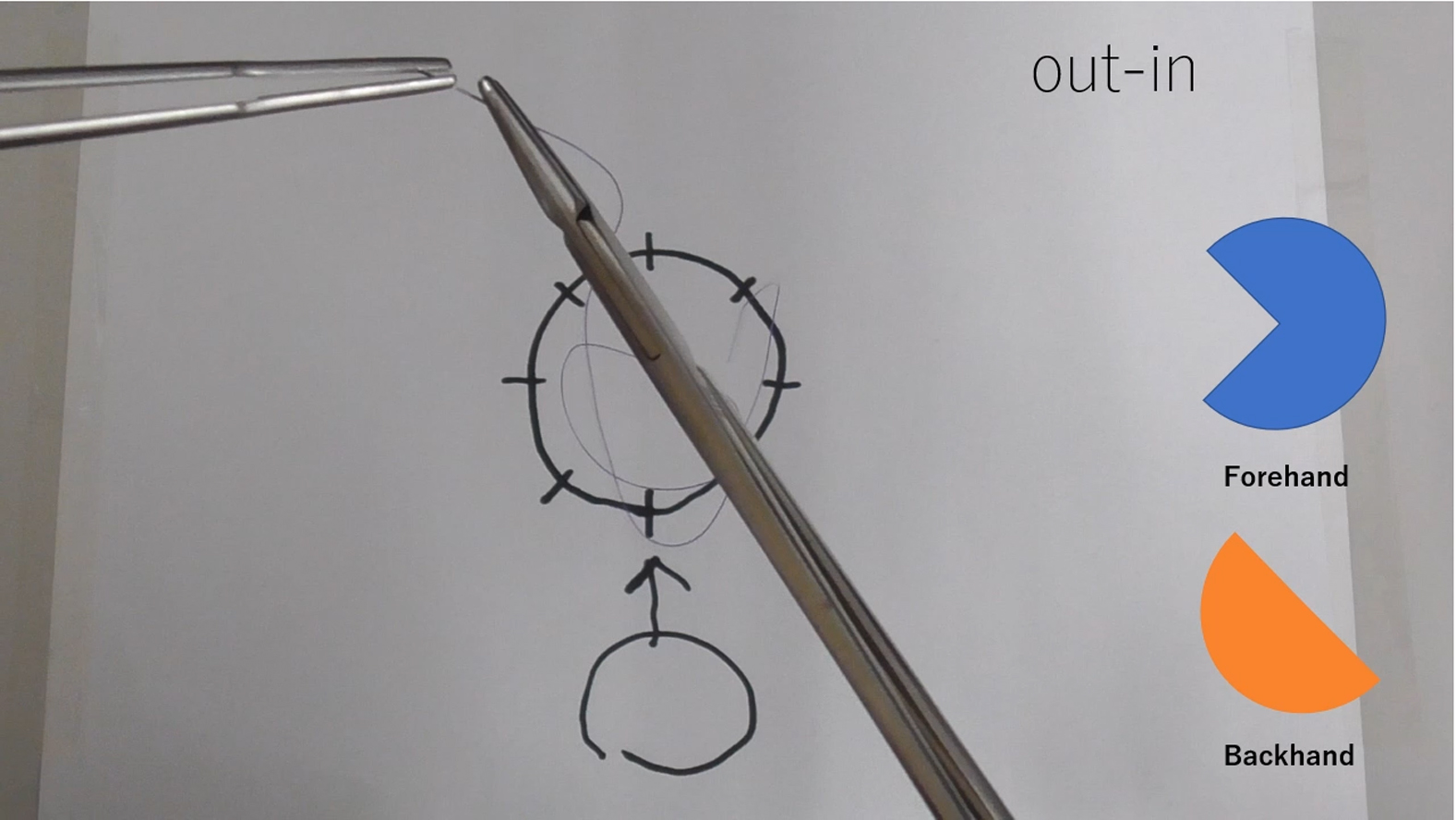

Supplement: Video 4 — Basic needle handling drill. Part 4. Video available at: https://www.jtcvs.org/article/S2666-2507(24)00274-8/fulltext. [file fx5.jpg]

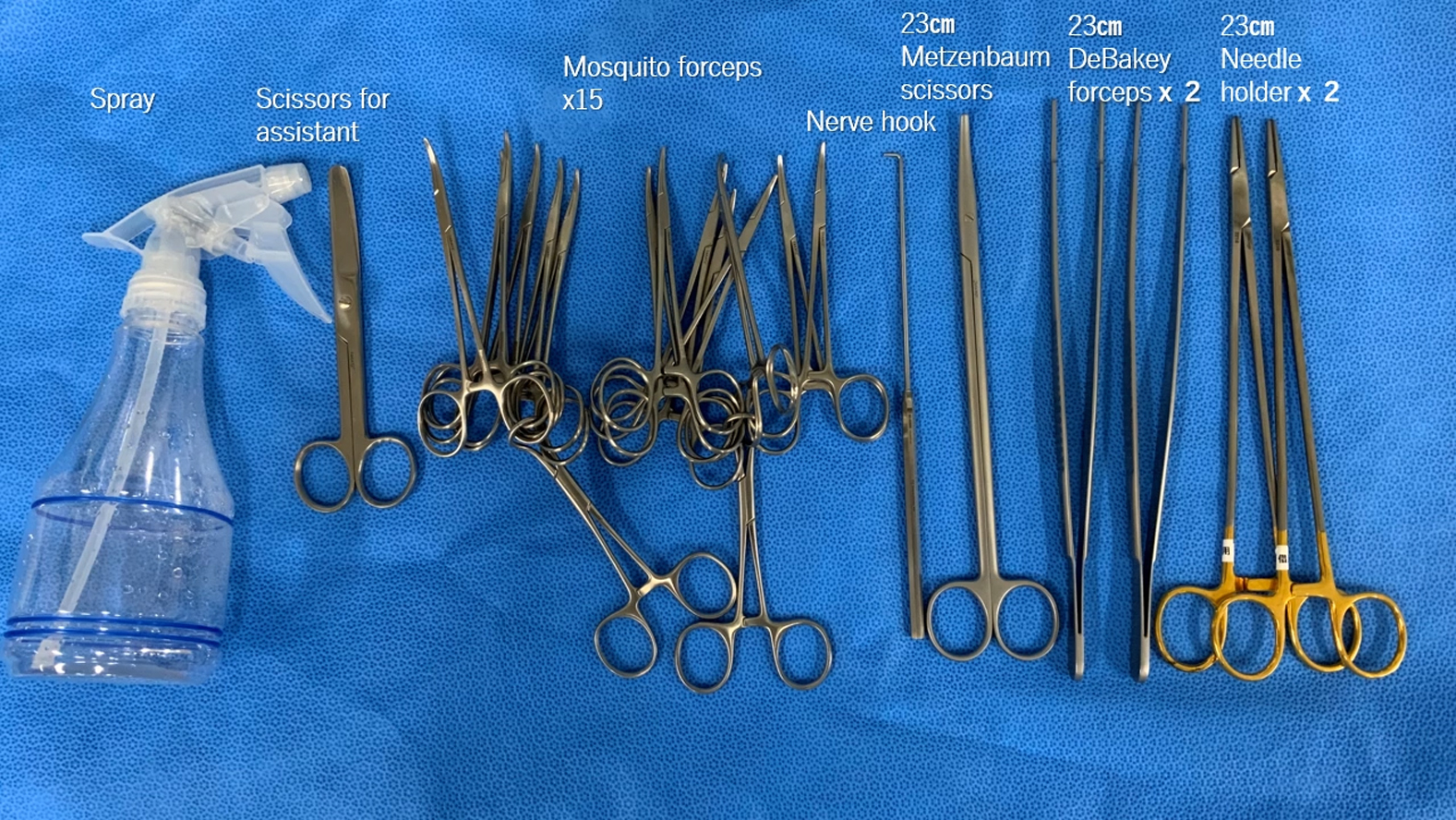

Supplement: Video 5 — Anastomosis drill. Part 1. Video available at: https://www.jtcvs.org/article/S2666-2507(24)00274-8/fulltext. [file fx6.jpg]

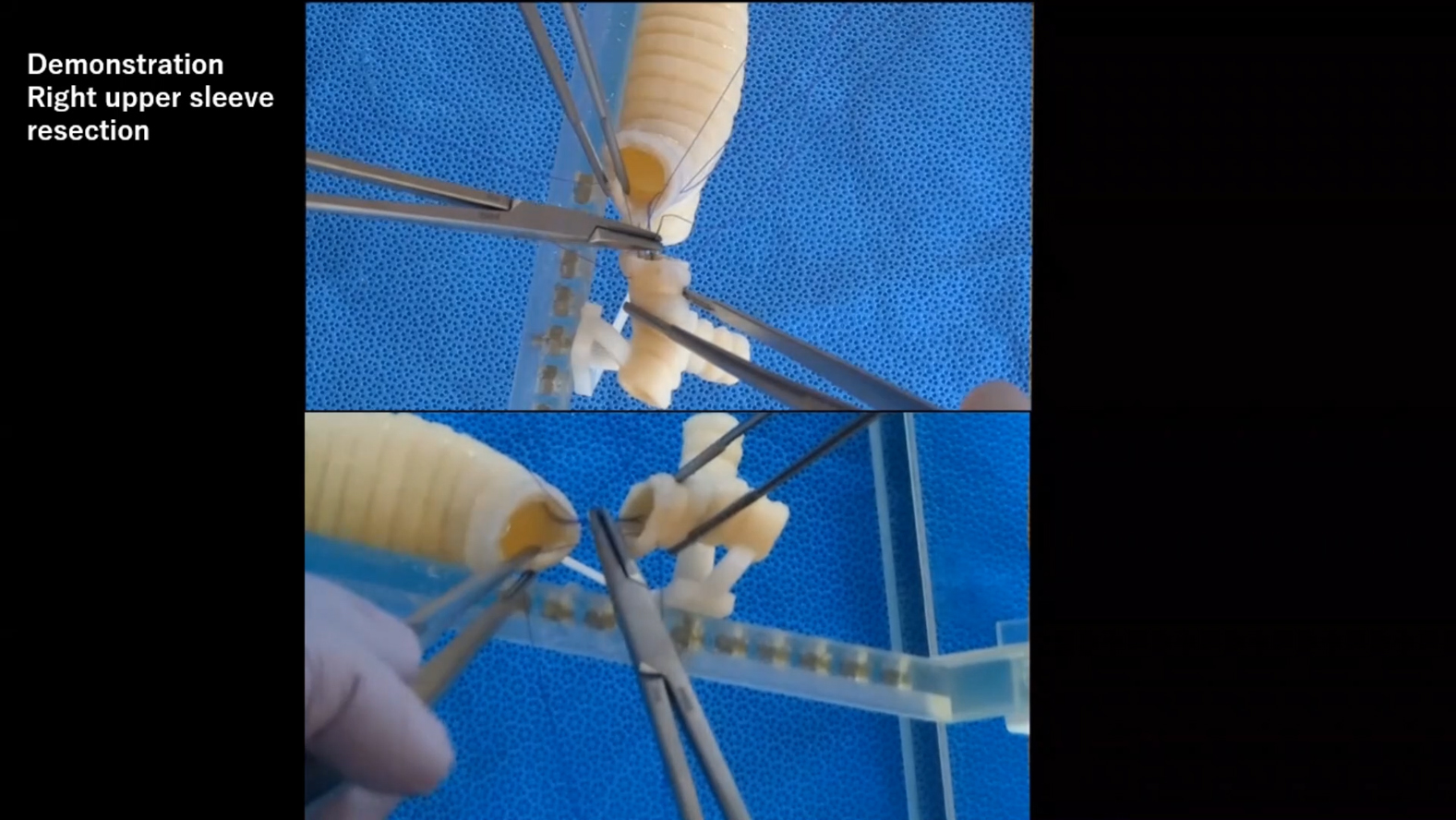

Supplement: Video 6 — Anastomosis drill. Part 2. Video available at: https://www.jtcvs.org/article/S2666-2507(24)00274-8/fulltext. [file fx7.jpg]

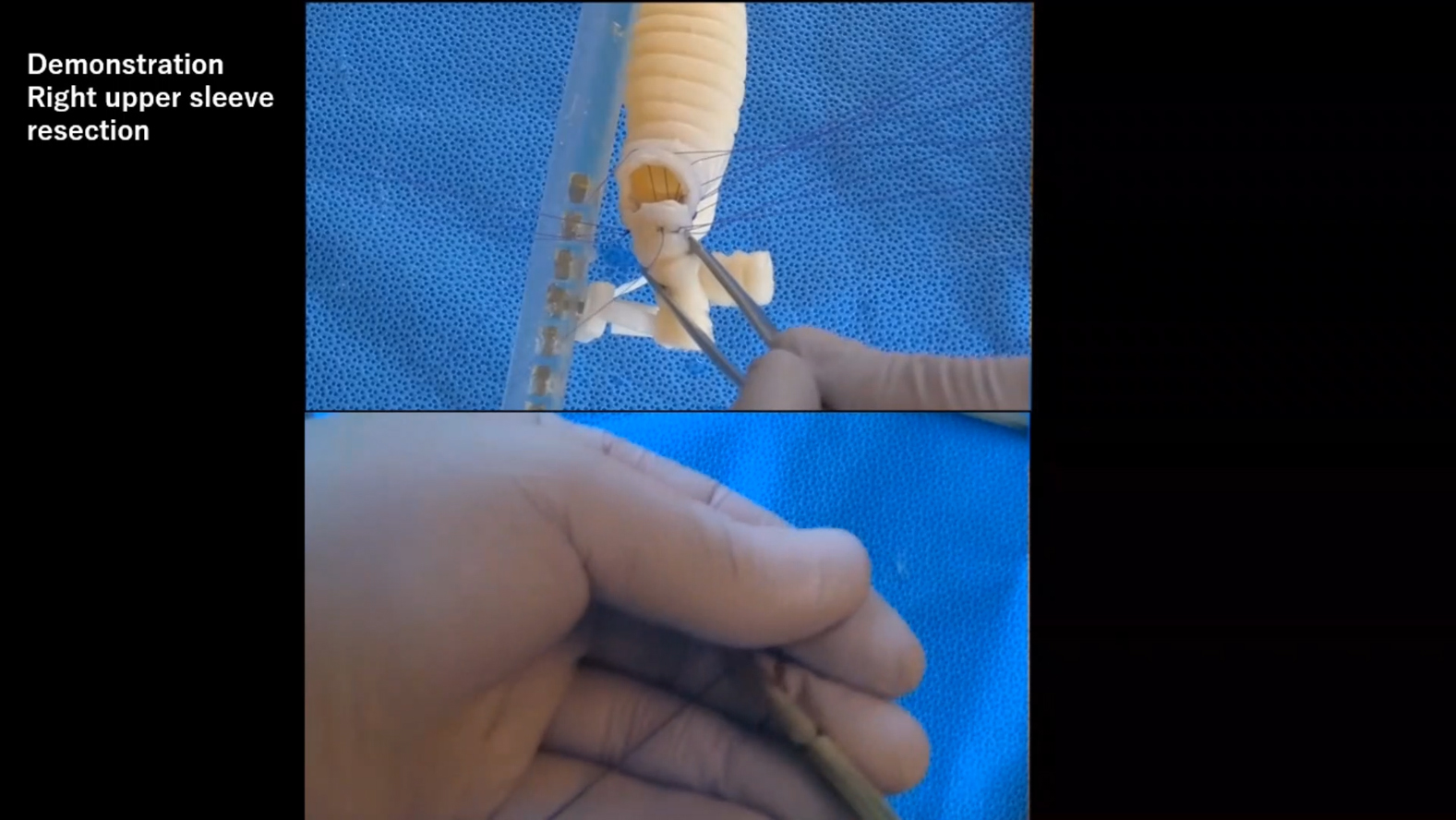

Supplement: Video 7 — Anastomosis drill. Part 3. Video available at: https://www.jtcvs.org/article/S2666-2507(24)00274-8/fulltext. [file fx8.jpg]

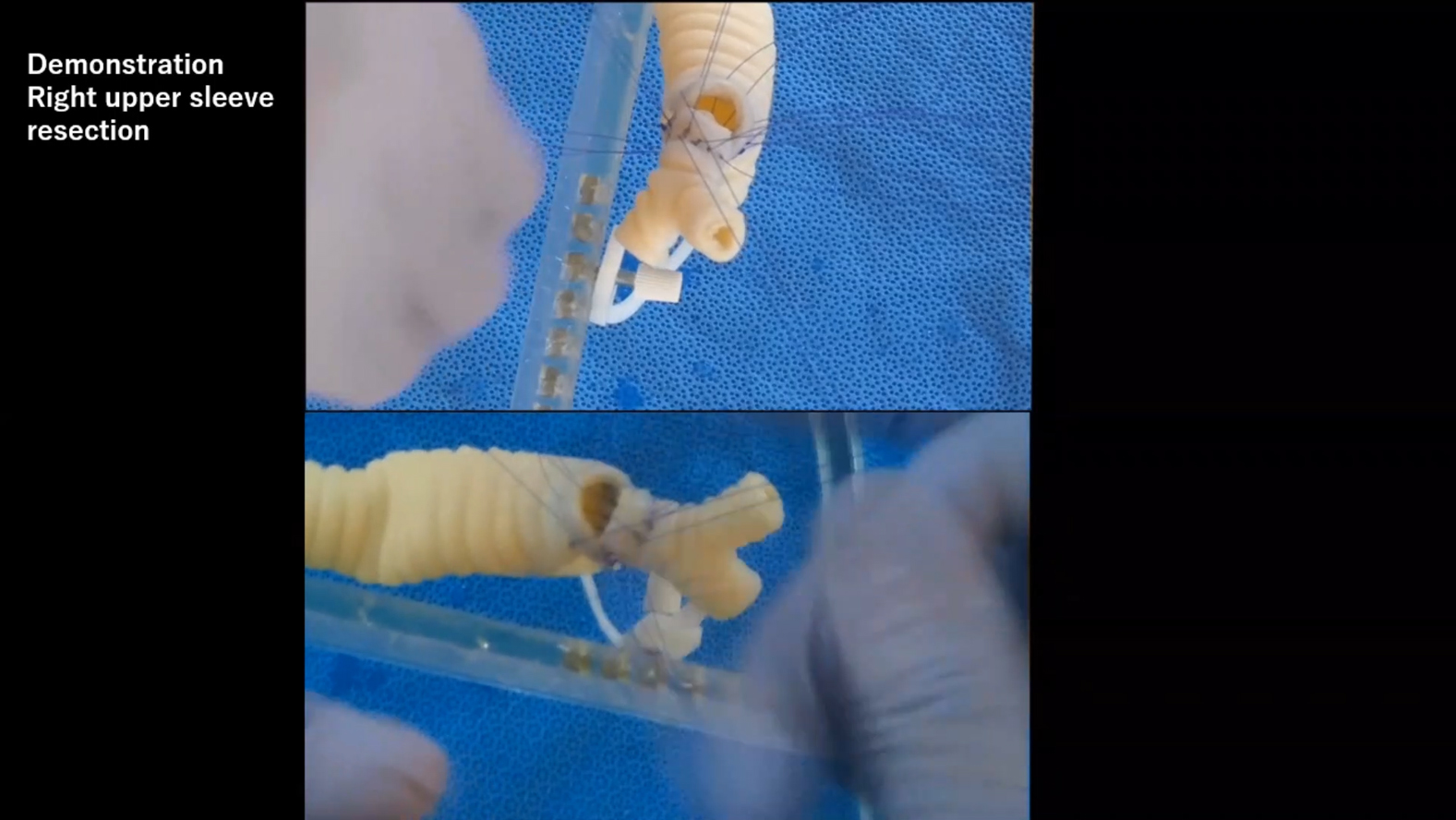

Supplement: Video 8 — Anastomosis drill. Part 4. Video available at: https://www.jtcvs.org/article/S2666-2507(24)00274-8/fulltext. [file fx9.jpg]
